# Finnish paramedics’ professional quality of life and associations with assignment experiences and defusing use – a cross-sectional study

**DOI:** 10.1186/s12889-021-11851-0

**Published:** 2021-10-05

**Authors:** Christoffer R. Ericsson, Hilla Nordquist, Veronica Lindström, Ann Rudman

**Affiliations:** 1 Faculty of Medicine, University of Helsinki, Helsinki, Finland; 2 Department of Healthcare, Arcada University of Applied Sciences, Jan Magnus Janssons plats 1, 00560 Helsinki, Finland; 3 South-Eastern Finland University of Applied Sciences, Kotka, Finland; 4 Department of Neurobiology, Care Sciences and Society, Section of Nursing, Karolinska Institutet, Stockholm, Sweden; 5 Department of Health and Welfare, Dalarna University, Falun, Sweden

**Keywords:** Emergency medical services, Compassion fatigue, Secondary traumatic stress, Compassion satisfaction, Burnout, psychological, Occupational stress

## Abstract

**Background:**

Paramedics experience traumatic events and social emergencies during assignments while also being subjected to verbal and physical threats. Consequently, they are at risk for burnout and secondary traumatic stress, factors inherent to professional quality of life. Defusing and peer-support potentially decrease such symptoms; however, perceived defusing needs and use are not always balanced. Our aim was to explore Finnish paramedics’ professional quality of life, using the Professional Quality of Life Scale, with associations to EMS assignment experiences as well as formal and informal defusing need and use over a 12-month period.

**Methods:**

A quantitative study of 257 Finnish paramedics using a cross-sectional design. Study outcomes were secondary traumatic stress (STS), compassion satisfaction (CS), and burnout (BO) scores using the modified 9-item Short Professional Quality of Life scale (ProQOL). Likert-type scales were used to collect participants’ recollections of assignment experiences and defusing from a 12-month period. Associations were explored using Spearman’s correlation coefficients.

**Results:**

Short ProQOL score medians were STS 4.00 (IQR 3), BO 6.00 (IQR 3) and CS 13.00 (IQR 3). STS and BO correlated to experiences of social emergencies and traumatic events while BO correlated to experiences of threat situations (*r* = 0.206, *p* = .001). Paramedics perceived a need for defusing in general associated with STS (*r* = 0.178, *p* < .001) and participated in informal defusing. Participation in defusing of any form did not associate with ProQOL scores.

**Conclusions:**

Finnish paramedics’ more frequent experiences of social emergencies, traumatic events, and paramedic-directed threat situations were associated with higher levels of STS and BO. STS was also associated with paramedics’ increased need for defusing and use of informal peer defusing, although neither STS, BO or CS scores associated to any defusing form. Managing paramedics STS and BO, while fostering CS, could therefore be a future research focus.

**Supplementary Information:**

The online version contains supplementary material available at 10.1186/s12889-021-11851-0.

## Introduction

Emergency Medical Services (EMS) or pre-hospital ambulance work is traditionally characterised as high-stakes and risky in varying and unpredictable conditions [1]. Although traditionally focusing on trauma and emergency situations, paramedics today also often assess and treat patients with non-acute presentations [2] and act as first-line support for patients in social emergencies [3]. Such patient groups associate with social vulnerability, isolation, homelessness, and lower quality of life [4] and often for them, EMS might be the only point of care [3]. Paramedics’ potential to intervene in elderly neglect, for example, is not insignificant, as non-acute EMS encounters constitute up to a third of all ambulance assignments [2] while out of all transports, frequent EMS callers account for up to 40% [4]. Concurrently, paramedics are increasingly subjected to threats from patients or bystanders, with a majority of EMS personnel, between 57 to 93%, having reportedly encountered physical and verbal abuse in their work during the last decade [5, 6]. The continuous imbalance of high-intensity and psychologically demanding assignments with more mundane, low-acuity experiences can affect paramedics’ professional quality of life [7, 8] as they often feel emotionally affected by patient encounters, which manifests in symptoms of traumatic stress [9, 10].

The emotional burden of being first responder increases the risk of paramedics becoming victims of secondary trauma, a form of stress from helping other suffering or traumatized persons [11]. This associates with a higher risk of compassion fatigue (CF) [6, 10, 12], a construct considered to consist of two dimensions, namely secondary traumatic stress (STS) and burnout (BO). The former, STS, is the consequence of caring professionals being exposed to the stress and trauma of others [13]. Together, these constructs form paramedics’ professional quality of life [14, 15]. The Professional Quality of Life Scale, originally developed by Figley and Stamm, is a widely applied measuring scale of professional quality of life [13].

Stressors increasing the risk of secondary trauma, both STS and BO, derive from feelings of hopelessness, associated commonly with paramedics’ assignment experiences involving, among others, death and drug overdoses [9, 16], as well as a sense of powerlessness in helping others in need, prevalent in cases of social distress [10, 17]. In addition, paramedics’ repeated non-acute encounters might cause professional role dissonance, in comparison to the more historical profile of paramedics as critical life-savers [7], with potential implications on paramedics’ compassion satisfaction (CS). CS is referred to as an emphatic engagement and rewarding pleasure of helping others, functioning thus as a balance against the negative effects stemming from CF [15]. Paramedics’ emotional suppression, a negative coping mechanism resulting from such frequent exposures to suffering, also associates to increased risk of cynicism, desensitisation and lower professional empathy [18, 19]. A sense of empathy, in turn, associates with lower levels of BO [20]. Consequently, long-term exposure to above-mentioned job-related stressors is reflected in paramedics’ more frequent turnovers and increased days of sick leave [21, 22]. As such, emotionally burdening experiences involving trauma and empathic suffering, which paramedics are exposed to during their on-call duty, on somewhat routine basis, has a remarkable potential to lowering paramedics’ professional quality of life through build-up of compassion fatigue or burnout, with, at times, unwanted or detrimental results [10, 12–15].

One approach to strengthen resilience and coping mechanisms and manage CF is formalised defusing: structured reflective meetings in a particular time and space, often led by trained personnel. However, evidence of the effect of defusing is inconclusive. The efficacy of formal peer defusing has been contested, partly showing no improvement or worsening symptoms of post-traumatic stress disorder (PTSD) and STS [23]. Meanwhile, some findings attest that formal debriefings potentially reduce the risk of BO among paramedic populations [24]. At the same time, informal defusing, characterised as non-structured ‘ad hoc’ peer-based reflective discussions around an event or assignment, might have positive effects on emotional processing through mechanisms strengthening resilience [25]; however the certainty of evidence is low [26]. All the while, EMS tradition and culture maintains a strong social desirability for peer approval and admitting support needs for work-related psychological burdens easily associates with stigmatism [12], raising the threshold for use of any form of defusing. This potentially leads to normalisation of paramedics’ STS and BO [8, 17] with detrimental effects on career longevity. Consequently, the meaningfulness of paramedics’ defusing use, in any form, could have implications for paramedics’ professional quality of life and career longevity, specifically in regards to lower incidences of burnout and secondary traumatic stress, resulting from availability for emotional unloading, as referenced by Cantu et al. [24, 25].

There is a need to further understand the degree to which paramedics’ assignment experiences, more specifically psychologically demanding experiences including suffering and trauma, with potential to cause compassion fatigue or burnout among paramedic professionals (henceforth referred to “experiences of EMS assignments”), as well as individual needs, and use of, formal and informal defusing associate with paramedics’ STS, BO and CS. To our knowledge, such relationships have not been investigated before. Our aim was therefore to explore Finnish paramedics’ professional quality of life and its possible associations with EMS assignment experiences as well as formal and informal defusing need and use over a 12-month period. We approached this through two research questions: 1) how do paramedics’ levels of STS, BO, and CS associate with memories of 12-month assignments of social emergencies, traumatic events, and paramedic-aimed threats, and 2) how do paramedics’ 12-month recalled use and perceived need for formal or informal debriefing associate with levels of STS, BO and CS.

## Methods

### Emergency medical services in Finland

Finnish paramedics are healthcare professionals recognised by the National Supervisory Authority for Welfare and Health in accordance with the Act on the Recognition of Professional Qualifications as stated in the Professional Qualifications Directive (2005/36/EC) and Finnish Health Care Act (1326/2010). Qualification requirements for EMS operative levels are as follows: Basic-Level Paramedics require a vocational degree as practical nurse with prehospital specialization, Basic-level Paramedic (equivalent to Emergency Medical Technician), firefighter or registered nurse. Requirements for Advanced-Level Paramedics are either a registered nurse qualification with advanced-level pre-hospital specialisation or an Emergency Care/Nursing dual Bachelor degree. Community Paramedics are Advanced-Level Paramedics, single-manned units usually dispatched to non-critical patients, while EMS supervisor units are often dispatched to high-acuity alarms requiring specialised care. Such units are also manned by Advanced-Level Paramedics with extensive pre-hospital experience, the EMS Supervisor having added operative responsibilities. While EMS units more often respond to all acuity levels (A-D) of assignments, other units handle exclusively non-acute (D) assignments or between-hospital transfers. The length of shifts varies depending on geography and service provider; traditional shift lengths for paramedics are usually 24- or 12-h, however shorter shifts are applied at times. Shift length corresponds to assignment prevalence, as non-acute assignments are typically more prevalent during evenings and nights [27].

### Study design and participants

We used a survey approach with a cross-sectional design. The study outcome was secondary traumatic stress (STS), burnout (BO), and compassion satisfaction (CS) scores collected using the 30-item Professional Quality of Life Scale R-IV (ProQOL). A quantitative recall-based questionnaire was used to collect participants’ approximated frequencies of psychologically demanding assignment experiences and defusing use from a 12-month period. The ProQOL Scale and the questionnaire were in Finnish and Swedish, as Finland is bilingual. Data was gathered in June 2019 during a 30-day period using an online questionnaire, distributed through three Finnish EMS social media groups, two of them closed to public viewing. The members count of each forum were between 281 and 4896. The questionnaire was subsequently further shared by users through social media. Participants were required to have EMS work experience for a minimum of 12 months as a Basic-Level Paramedic, Advanced-Level Paramedic, Community Paramedic, or EMS Supervisor. Non-operative administrative personnel, first responders, and volunteer firefighter units were excluded due to high variations in EMS alarms among such units.

### Measures

The questionnaire (Additional file 1) included six socio-demographic and job-related variables (gender, age, paramedic work experience, EMS operative level, assignment acuity level response, shift length), the 30-item ProQOL R-IV Scale, and eighteen Likert-type items for participants’ recalled experiences of EMS assignments, categorised into three main categories — social emergencies, acute traumatic events, and paramedic-directed threat situations — further specified into fifteen specific variables. Four items measured participants’ perceived need for and use of workplace defusing, differentiated into organised (i.e. a meeting constrained to a specific time and place, often mandatory, moderated by defusing-trained personnel) or informal (i.e. non-mandatory peer discussion of a specific assignment or situation, not bound to a specific time and place and not moderated), as well as the perception of the positive effects on paramedics’ well-being from participation in defusing. The recall period was 12 months [28]. The 6-point ordinal Likert-scales ranged from 1 (Never or Completely Disagree) to 6 (Very Often or Agree Completely). In total, 59 items were collected.

The Professional Quality Of Life R-IV Scale, Finnish and Swedish versions [29], was used to collect STS, BO, and CS data. The ProQOL Scale is a widely used and popular scale for measuring CF and CS, with several published studies having used the scale in a variety of, among others, healthcare contexts [13, 30]. According to the framework of the ProQOL, the overarching concept of CF is composed of STS and BO, with the second concept being CS [13, 31]. The original ProQOL Scale is a self-report scale, consisting of the three subscales (STS, BO and CS) with 10 items per subscale. The items assess frequency of symptoms using a five-point Likert Scale [30] Although the ProQOL Scale is not without controversy, mainly regarding the psychometric properties and validity, there is enough support for its use both for scoring bifactorial CF and CS [31] and also three-factor structure, STS, BO and CS [30].

Based on a small-sample pilot study, additions to translations and item definitions were appended, but no original language or items were altered. Internal consistency analysis on the 30-item ProQOL R-IV (30 items) subscales showed Cronbach’s alpha .80 for STS, .70 for the BO scale and .89 for the CS scales, similar to previous reported findings in which issues with the BO scale for healthcare populations have been raised [30]. To our knowledge, no consistency analyses of the Finnish or Swedish ProQOL have been reported. A confirmatory factor analysis of the ProQOL R-IV Scale, using jamovi (v 1.2.27), showed borderline model fit. As such, supported by a scoping review, we modified the scales post-hoc into the more robust 9-item Short ProQOL Scale [32], resulting in better model fit, with similar internal consistency values: .74 for STS, .80 for BO, and .85 for SC (Additional file 2). Mean ProQOL Scale scores were reported in raw scores, with scales ranging between 3 to 18. Score reference groupings were not used due to scale modifications from the original scale.

### Data analysis

Statistical analyses were done using SPSS for Windows, v 26. The alpha level was set at 0.05 for statistical significance. Kolmogorov-Smirnov tests showed none of the ProQOL subscales were normally distributed (*p* < 0.05). Therefore, non-parametric analyses, Spearman’s correlations, and Kruskal-Wallis [33] were used to explore correlations between ProQOL scores, participants’ demographic data, and recalled item means while predictions were explored through linear regressions. Considering the non-symmetrical distributions and Likert-type ordinal data, medians were used [34, 35]. Missing data were found with Little’s MCAR Test *p* > 0.05, therefore list-wise deletion was used.

## Results

The population (*N* = 257) median age was 32 (IQR 9) years with 54% of the population being female. EMS work experience ranged from one to 35 years, median 7 (IQR 7) years. Age correlated strongly to EMS work experience (*r* = 0.658; *p* < 0.001). In total, 90.6% worked either 24 h or 12 h work shifts and the majority (175, 68.4%) worked as Advanced-Level Paramedics. For detailed demographics, see Additional file 3.

**Fig. 1:**
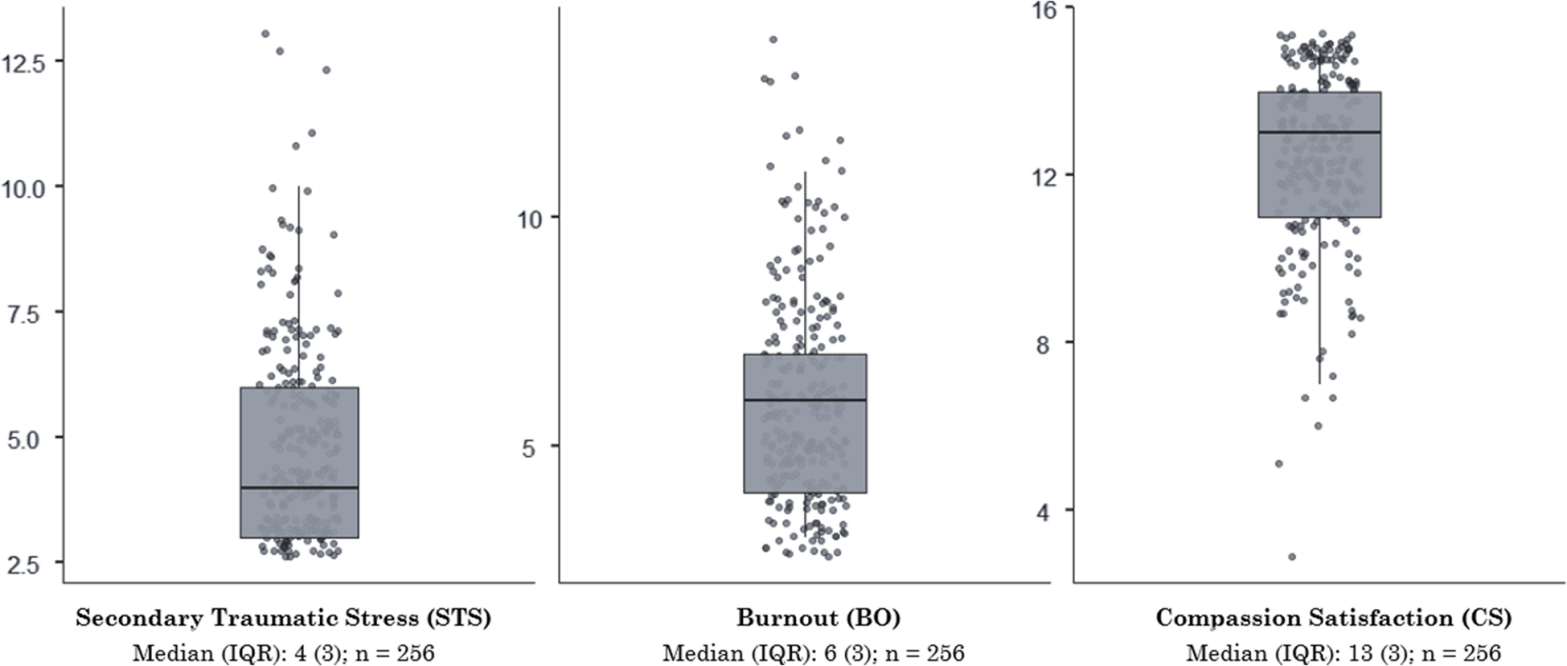
Short ProQOL Scale (9-item) score distributions in population

Short ProQOL Scale medians were: STS 4.00 (IQR 3), BO 6.00 (IQR 3), and CS 13.00 (IQR 3) (Fig. 1). No significant ProQOL scale differences were found between socio-demographic or population variables (Additional file 4). CS correlated negatively to both STS (*r* = − 0.296; *p* < 0.01) and BO (*r* = − 0.417; *p* < 0.01) (Additional file 5).

Paramedics’ experiences of social emergencies during EMS assignments had a score median of 5.00 (IQR 2), with 69% having experienced social emergencies either often or very often within the last 12-month period. Traumatic experiences during EMS assignments had a score median of 3.00 (IQR 1), with 76% experiencing trauma seldom or sometimes within the last 12 months. Paramedic-directed threat situations during EMS assignments had a score median 4.00 (IQR 1), with 74% experiencing physical or verbal threats seldom or sometimes within the last 12 months (Table 1). EMS assignment experiences involving drugs and/or alcohol use and social exclusion were most common. More detailed distributions are found in Additional file 6.

**Table 1:**
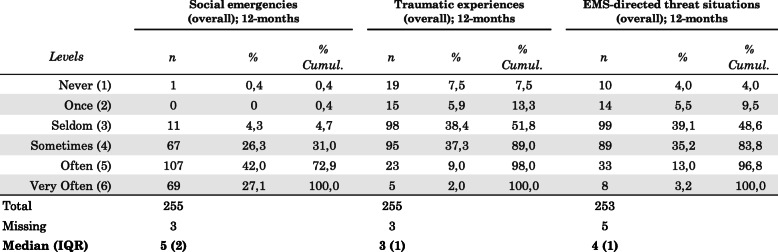
EMS assignment (overall) experience levels

Paramedics seldom (87; 33.7%) or sometimes (77, 29.8%) felt a need for any form of defusing after an EMS assignment (median score 3.00 ± 1.226). Participations in formal defusing were altogether fewer (median score 1.00; IQR 1) compared to participation in informal defusing (median score 3.00; IQR 2). The majority of respondents, 67.5% (174), had never participated in formal defusing, while 21.3% (55) had participated only once. However, 40.7% (105) agreed that taking part in any form of defusing had a positive effect on their well-being (median score 4.00; IQR 2). For a more detailed distribution, see Additional file 7.

### EMS assignment experiences and paramedics’ professional quality of life

**Fig. 2:**
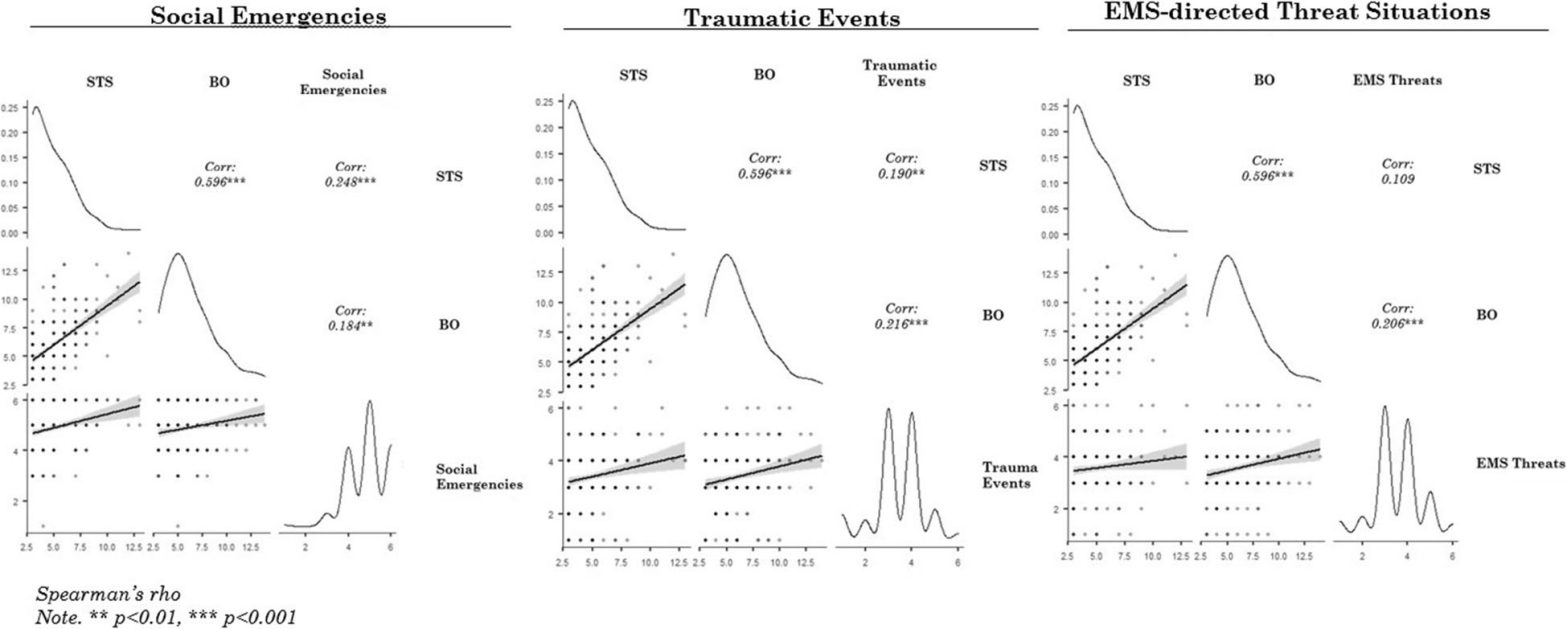
EMS assignment (overall) experiences associations to ProQOL Scores

Higher levels of social emergency experiences during EMS assignments correlated with paramedics’ STS (*r* = 0.220) and BO (*r* = 0.187) levels, as did experiences of traumatic events with STS (*r* = 0.168) and BO (*r* = 0.205). Experiences of paramedic-directed workplace violence correlated only with BO (*r* = 0.189). CS did not correlate significantly with any of the overall experience categories (Fig. 2).

Exploring specific EMS assignment experiences of social emergencies, STS correlated strongest with paramedics’ experienced need for elderly protective social services (*r* = 0.242), drugs and/or alcohol use (*r* = 0.214), and social exclusion (*r* = 0.208) while BO correlated most prominently with experiences of social exclusion (*r* = 0.185) and assignments involving violence (*r* = 0.176). While both physical and verbal paramedic-directed threats correlated with BO (*r* = 0.153 and *r* = 0.192, respectively), physical threats also correlated negatively to paramedics’ CS (*r* = − 0.125) (Additional file 8).

### Defusing and paramedics’ professional quality of life scores

Paramedics’ increased need for post-assignment defusing in general associated with higher STS (*r* = 0.178). Paramedics’ more frequently perceived need for forms of post-assignment defusing associated with more frequent participation in forms of informal defusing (*r* = 0.513), which in turn associated with more frequent participation in formal defusing (*r* = 0.255). While paramedics’ subjective perceptions of positive effects from attending defusing was associated with both lower BO (*r* = −.162) and higher CS (*r* = 0.221), actual participation in any form of defusing did not significantly correlate with BO, STS, or CS (Table 2).

**Table 2:**
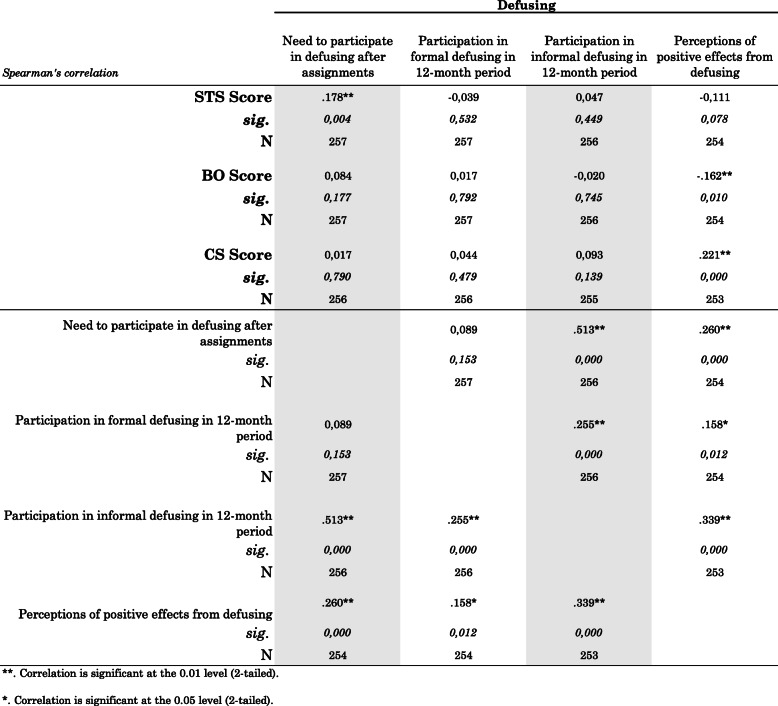
Paramedics’ defusing need and participation associations to 9-item Short ProQOL scores

## Discussion

The study set out to explore how psychologically demanding and traumatic EMS assignment experiences, as well as defusing use, associated with Finnish paramedics’ STS, BO and SC levels, measured using the 9-point modified ProQOL Scale. Although the results were in line with previous studies of paramedic populations, variations in paramedics’ BO and STS measurements could be attributed to differences in use of measuring instrument and a generally varying definition of ‘paramedic’ and ‘first responder’ [1, 11, 36].

### Associations between EMS dispatch experiences and paramedics’ professional quality of life

Paramedics generally recalled more frequent experiences of social emergencies compared to experiences of traumatic events and paramedic-focused threats, with the latter recalled in moderate amounts. Experiences of social emergencies and acute traumatic events both associated with increased STS and BO, while more frequent paramedic-focused threats associated only with higher BO. Although associations were weak and assignment experiences alone do not explain paramedics’ levels of BO or STS, they still suggest that routine but psychologically demanding experiences of social emergency assignments associate with paramedics’ STS and BO. Exposures to routine alarms can lead to varying stress responses between paramedics attending seemingly mundane assignments [12], emphasising the undetected psychological demands of such routine experiences on healthcare personnel. A secondary finding was a moderate association between paramedics’ recall of a need for child protection and a need for elderly protective social services, non-related experiences both involving an empathetic recognition action by paramedics. This is pertinent considering the negative relationship between empathy and BO [20].

Furthermore, the literature on paramedics’ mental health has a rather trauma-centred perspective, often looking specifically at relationships between exposure to traumatic events. This study, in turn, attempted to observe a wider variety of routine experiences paramedics encounter. It has been hypothesised that paramedics potentially prefer more “prized and valued” trauma care over routine caring work, due to a more limited emotional connection [18]. Such emotional suppression from repeated exposures associates with increased cynicism, emotional desensitisation, and lower empathy among paramedics [18, 19], which is especially relevant since paramedic students show declining empathy levels early during their studies, possibly resulting from exposure to the ‘reality of praxis’ [37]. This is a growing concern, since CF could have potentially detrimental effects on both paramedics’ career longevity and also patient safety [38, 39]. The secondary finding, noted above, calls for a discussion on the effects of BO on such empathic decision reactions [20]. The altruistic value of caring for others and the excitement of the unpredictable are noted as two main motivations for choice of a paramedic career [18]. It would be reasonable to suggest that these motivators link to CS, potentially building resilience against STS and BO [14]. As a conclusion, upholding and sustaining altruistic motivators may potentially further paramedics’ CS while managing STS during and before clinical work, with benefits for paramedics’ career longevity.

### Associations between defusing and paramedics’ professional quality of life scores

Our results suggested that participants’ need for post-assignment defusing correlated with STS. However, while more positive perceptions of defusing did associate with CS and BO, actual participation in defusing did not show significant associations with STS, BO, or CS levels. These results underscore the mixed effects of defusing [26]. While this study explored only participants’ attendance of defusing sessions, the quality and content of attended sessions would be of value in understanding their effect. Moreover, whether the low prevalence of formal defusing reflected paramedics’ attitudes towards support seeking or a high threshold for defusing activation in EMS organisations, remained unknown. A study by Murray et al. (2018) on moral injury among medical students in pre-hospital emergency medicine suggested that frequent social support from colleagues and family can potentially have positive outcomes on students’ recovery from unresolved feelings of shame and guilt among others [40]. There is no reason to believe that these results are not also applicable to CF. Other theories suggest that paramedics’ generally lower STS and BO scores are partly explained by the need to fit in socially, a ‘culture of managing’ prominent in paramedic and acute healthcare cultures, leading to positive response biases [11, 12]. EMS cultures are historically hard and fast, with an over-burdened and fatigued personnel, where admitting to mental health issues can provoke an almost stigmatising effect, leading to detrimental coping strategies such as avoidance and depression [12, 18]. As such, emphasis of informal and low-threshold defusing and acknowledgement of mental burdens in frontline healthcare workplace cultures and during the educational period seems relevant.

Paramedic work involves psychologically challenging assignments alongside responsibilities not always innately connected to the perceived role as an acute life-saver. This demanding and empathic work clearly associates with paramedics’ higher STS and BO. There are signs of the mitigating effect of CS on BO and STS with resilience strengthening coping mechanisms, increasing professional retention and job satisfaction [14, 41]. The need to understand how mundane but psychologically demanding work potentially affects paramedics’ STS and BO while strengthening CS was the focus of this study. This is a relevant issue, especially during the ongoing COVID-19 pandemic, which puts higher than usual psychological pressure on frontline healthcare personnel on a continuous basis, affecting their well-being [42]. In addition to the unique macro-level perspective on the variations of paramedic assignments, the focus on defusing furthers the discussion of acute care professionals’ often private and undisclosed need for peer support to manage the caring work they do. Additionally, as this study was conducted before the onset of the COVID-19 pandemic, the results mirror what could be considered ‘normal’ Finnish EMS working conditions, thus helping to identify the degree to which such work potentially corresponds to paramedic and EMS personnel resilience, well-being, job satisfaction, and ultimately career longevity after the pandemic has passed.

### Limitations

This study is not without limitations. Social media-based data collection potentially led to sampling bias, apparent in the skewness towards younger participants. Omissions of background, geographic, and educational factors also possibly concealed confounding factors, which could explain the results further. The reliability of recall-based collection methods has also been disputed, with reported errors in details from longer recall periods. The method, however, sheds a unique and relevant perspective, allowing for the possibility to explore paramedics’ perceptions of annual assignments on an individual experiential level. The study also had a comparably good sample size with wide demographic and occupational variations, allowing a representation of the Finnish paramedic population and aiding in generalisability of the results. However, due to the self-report methodology, there were a few missing values. The post-hoc modification to Short ProQOL Scale resulted in methodological strengths and limitations; while the scale had a better model fit, the novelty of the scale made comparison with previous results challenging. However, the macro-level approach merited the use of recall-based methodology and the more reliable ProQOL scale was merited due to issues raised in the original specifically among frontline healthcare populations. Lastly, the use of a cross-sectional observational study design does not allow any conclusions of causality or temporal relationships between the exposure and outcomes and any associations found may be difficult to interpret, as potential confounding variables cannot be entirely ruled out [43].

## Conclusions

Finnish paramedics’ frequent psychologically demanding experiences of social emergencies and traumatic situations associated with higher levels of STS and BO. STS associated with an increased need for defusing and the use of informal peer-support defusing; however, no defusing form associated with STS, BO or CS scores. Further studies could explore what role paramedic education has for managing STS and BO while fostering CS and also whether, for instance, emergency care/nursing dual bachelor-degree students develop an earlier or stronger need to ‘manage the work’ compared to other healthcare students.

## Supplementary Information

**Additional file 1.** Questionnaire.

**Additional file 2.** ProQOL Scales Confirmatory Factor Analysis.

**Additional file 3.** Population Demographics.

**Additional file 4.** Short ProQOL (9-item) Scale score distributions across population parameters.

**Additional file 5.** Short ProQOL (9-item) scales correlations.

**Additional file 6.** EMS assignment (specific) experience levels.

**Additional file 7.** Paramedics’ defusing need and participation.

**Additional file 8.** EMS assignment (specific) experiences associations to ProQOL scores.

## Data Availability

The datasets used and analysed during the current study are available from the corresponding author on reasonable request.
