# Supplementary material for: Finnish paramedics’ professional quality of life and associations with assignment experiences and defusing use – a cross-sectional study
Source: BMC Public Health. 2021 Oct 5;21:1789. doi: 10.1186/s12889-021-11851-0 (PMC8490964; doi:10.1186/s12889-021-11851-0)
Supplement: Supplementary file 1 — Additional file 1. Questionnaire. [file 12889_2021_11851_MOESM1_ESM.docx]

**Additional** **file 1. Questionnaire**

**Demographic (6 items)**

Gender (Male/Female/Other)

Age (years)

EMS Operative Level (Basic-Level/Advanced-Level Paramedic/Community Paramedic/EMS Supervisor)

EMS Work experience (years)

Shift Length (24h/12h/8h/Other)

Acuity Level Response (All Levels, Only D-Level, Only Hospital Transfers)

**Professional Quality of Life Scale (Likert Scale 1-5; 30-items), Scale: 1 (Strongly Agree) to 5 (Strongly Disagree).**

1. I am happy.

2. I am preoccupied with more than one person I help.

3. I get satisfaction from being able to help people.

4. I feel connected to others.

5. I jump or am startled by unexpected sounds.

6. I feel invigorated after working with those I help.

7. I find it difficult to separate my personal life from my life as a helper.

8. I am losing sleep over traumatic experiences of a person I help.

9. I think that I might have been “infected” by the traumatic stress of those I help.

10. I feel trapped by my work as a helper.

11. Because of my helping, I have felt “on edge” about various things.

12. I like my work as a helper.

13. I feel depressed as a result of my work as a helper.

14. I feel as though I am experiencing the trauma of someone I have helped.

15. I have beliefs that sustain me.

16. I am pleased with how I am able to keep up with helping techniques and protocols.

17. I am the person I always wanted to be.

18. My work makes me feel satisfied.

19. Because of my work as a helper, I feel exhausted.

20. I have happy thoughts and feelings about those I help and how I could help them.

21. I feel overwhelmed by the amount of work or the size of my caseload I have to deal with.

22. I believe I can make a difference through my work.

23. I avoid certain activities or situations because they remind me of frightening experiences of the people I help.

24. I am proud of what I can do to help.

25. As a result of my helping, I have intrusive, frightening thoughts.

26. I feel “bogged down” by the system.

27. I have thoughts that I am a “success” as a helper.

28. I can't recall important parts of my work with trauma victims.

29. I am a very sensitive person.

30. I am happy that I chose to do this work.

**EXPOSURE TO SOCIAL EMERGENCIES** **during the last 12 months**

**Instructions: Please rate the following statements on a scale of 1 (None) to 6 (Very often). If you have never experienced situations of social distress, you would circle 1.**

***Social emergency:*** *Witnessed or experienced incidents involving either clearly long-term social emergencies; elderly or social neglect, loneliness, homelessness, substance or alcohol addiction or acute social emergencies; child abuse and child protection needs, acute mental problems or other situations requiring acute need for social service support.*

Experience of: Loneliness or loss of social support networks

Homelessness

Substance or alcohol abuse

Child protection needs

Elderly protection needs

Elderly social needs

OVERALL SOCIAL EMERGENCIES

**EXPOSURE TO TRAUMATIC EVENTS during the last 12 months**

**Instructions: Please rate the following statements on a scale of 1 (None) to 6 (Very often). If you have never experienced traumatic events, you would circle 1.**

Experience of: Resuscitation of an infant or a child

Critically ill infant or child

Serious trauma involving adult or child patient

Traumatic patient suicide

Traumatic death

Act of violence (not directed at EMS personnel)

Mass casualty incident

Accident involving EMS personnel

OVERALL TRAUMATIC EVENT

***Traumatic event:*** *A witnessed or experienced incident that has clearly caused you some level of emotional, spiritual, or psychological harm. You have considered the distressing event threatening, anxious or frightening and normal coping methods have not felt sufficient.*

**EXPOSURE TO PHYSICAL OR VERBAL THREATS TOWARD PARAMEDICS during the last 12 months**

**Instructions: Please rate the following statements on a scale of 1 (None) to 6 (Very often). If you have never experienced situations of physical or verbal abuse, you would circle 1.**

Experience of: Physical threat towards yourself or colleague

Verbal violence towards yourself or colleague

OVERALL PARAMEDIC-DIRECTED THREATS

**DEFUSING USE AND NEED**

**Instructions: please answer the following questions on a scale of 1 (None) to 5 (Very often) – if you have never felt the need to participate or have never participated in defusing, you would circle 1.**

Have you **felt a need** to participate in formal (3) or informal defusing (4) during your EMS shifts?
(Scale: 1-6, Cannot Say)

Have you during the last 12 months, **participated** in formal defusing during your EMS work?
(Scale: 1-6, Cannot Say)

Have you during the last 12 months **participated** in informal defusing during your EMS work?
(Scale: 1-6, Cannot Say)

Have you felt that using any above defusing forms **has had a positive effect** on you?
(Scale: 1-6, Cannot Say)

*(3)* ***Organised defusing:*** *A defusing/debriefing system clearly organised by your employer and activated using clearly stated guidelines and protocol, managed and moderated by defusing-trained personnel. Involves exclusively only personnel affected by the situation. Often mandatory to attend for all involved after the relevant events. Example: a defusing session activated by shift supervisors or defusing-trained personnel on call.*

*(4)* ***Informal defusing:*** *A defusing/debriefing that does not follow any set guidelines, protocols or limitations. May or may not involve all personnel affected but not exclusively. No defusing-trained personnel necessary to moderate. Example: An informal discussion at base after the call with colleagues or team members or a gathering with no formal discussion structure.*
