# Supplementary figures and images for: Finnish paramedics’ professional quality of life and associations with assignment experiences and defusing use – a cross-sectional study
Source: BMC Public Health. 2021 Oct 5;21:1789. doi: 10.1186/s12889-021-11851-0 (PMC8490964; doi:10.1186/s12889-021-11851-0)

**Additional** **file 2. ProQOL Scales Confirmatory Factor Analysis Model Fit**

**
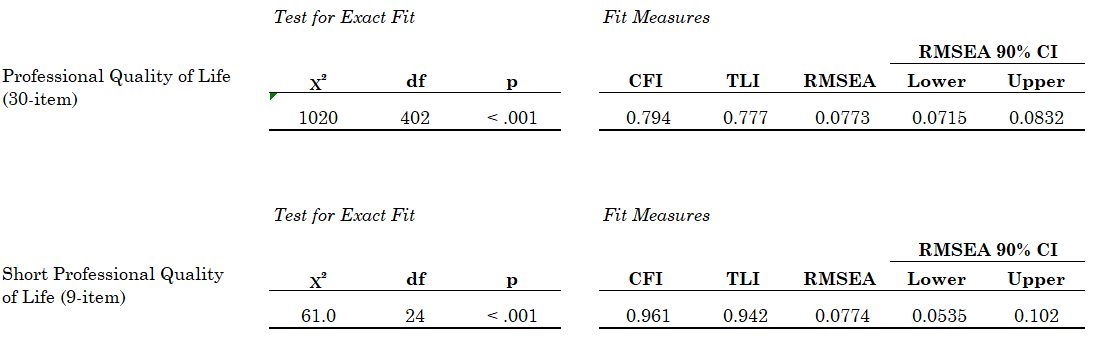
**

Supplement: Supplementary file 2 — Additional file 2. ProQOL Scales Confirmatory Factor Analysis. [file 12889_2021_11851_MOESM2_ESM.docx]

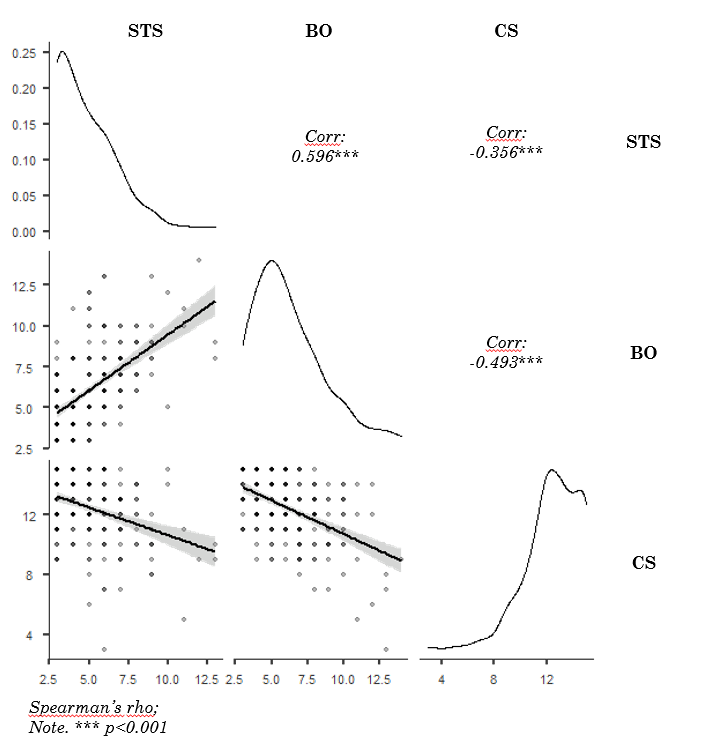


**Additional** **file 5. Short ProQOL (9-item) scales correlations**

Supplement: Supplementary file 5 — Additional file 5. Short ProQOL (9-item) scales correlations. [file 12889_2021_11851_MOESM5_ESM.docx]
