# Supplementary material for: Finnish paramedics’ professional quality of life and associations with assignment experiences and defusing use – a cross-sectional study
Source: BMC Public Health. 2021 Oct 5;21:1789. doi: 10.1186/s12889-021-11851-0 (PMC8490964; doi:10.1186/s12889-021-11851-0)
Supplement: Supplementary file 3 — Additional file 3. Population Demographics. [file 12889_2021_11851_MOESM3_ESM.docx]

| **Category** | **Class** | **n (%)** |
| --- | --- | --- |
| Gender *(n=257)* | Male | 118 (45,9%) |
|  | Female | 139 (54,0%) |
| Age, years *(n=254)* | 20 - 25 | 33 (13,0%) |
|  | 26 - 30 | 65 (25,6%) |
|  | 31 - 35 | 76 (29,9%) |
|  | 36 - 40 | 39 (15,4%) |
|  | 41 - 45 | 27 (10,6%) |
|  | 46 - 50 | 8 (3,1%) |
|  | 51 - 55 | 5 (2,0%) |
|  | > 55 | 1 (0,4%) |
| Paramedic Work Experience, years *(n=246)* | 1 - 3 | 47 (19,1%) |
|  | 4 - 6 | 67 (27,2%) |
|  | 7 - 9 | 46 (18,7%) |
|  | 10 - 12 | 30 (12,2%) |
|  | 13 - 15 | 26 (10,6%) |
|  | 16 - 18 | 8 (3,3%) |
|  | 19 - 21 | 12 (4,9%) |
|  | 22 - 24 | 4 (1,6%) |
|  | 25 - 35 | 6 (2,4%) |
| Operative EMS Level *(n=256)* | Basic-Level Paramedic | 60 (23,4%) |
|  | Advanced-Level Paramedic | 175 (68,4%) |
|  | Community Paramedic | 3 (1,2%) |
|  | EMS Supervisor Unit | 18 (7,0%) |
| Shift Length Type *(n=256)* | 24h | 122 (47,7%) |
|  | 12h | 110 (42,6%) |
|  | 8h | 2 (0,8%) |
|  | Other | 22 (8,6%) |
| EMS Dispatch Acuity  *(n=257)* | All Dispatch Acuities | 250 (97,3%) |
|  | Non-Acute Dispatch Acuity Only | 2 (0,8%) |
|  | Between-Hospital Transports Only | 5 (1,9%) |

**Additional** **file 3: Population Demographics**
