# Supplementary material for: Finnish paramedics’ professional quality of life and associations with assignment experiences and defusing use – a cross-sectional study
Source: BMC Public Health. 2021 Oct 5;21:1789. doi: 10.1186/s12889-021-11851-0 (PMC8490964; doi:10.1186/s12889-021-11851-0)
Supplement: Supplementary file 4 — Additional file 4. Short ProQOL (9-item) Scale score distributions across population parameters. [file 12889_2021_11851_MOESM4_ESM.docx]

**Additional** **file 4. Short ProQOL (9-item) Scale score distributions across population parameters**
