# Supplementary material for: Finnish paramedics’ professional quality of life and associations with assignment experiences and defusing use – a cross-sectional study
Source: BMC Public Health. 2021 Oct 5;21:1789. doi: 10.1186/s12889-021-11851-0 (PMC8490964; doi:10.1186/s12889-021-11851-0)
Supplement: Supplementary file 8 — Additional file 8. EMS assignment (specific) experiences associations to ProQOL scores. [file 12889_2021_11851_MOESM8_ESM.docx]

**Additional** **file 8. EMS assignment (specific) experiences associations to ProQOL scores**
